# First Genome Sequences of Dengue Virus Strains Isolated during the First DENV-4 Outbreak in São Paulo, Brazil

**DOI:** 10.1128/genomeA.00340-17

**Published:** 2017-07-13

**Authors:** Ayda Susana Ortiz-Baez, Danielle Ferreira Silva, Daniel Ferreira, Paolo M. Zanotto

**Affiliations:** Laboratory of Molecular Evolution and Bioinformatics, Department of Microbiology, Institute of Biomedical Sciences, University of São Paulo, São Paulo, Brazil

## Abstract

Dengue virus (DENV) is an arbovirus belonging to the genus *Flavivirus*, family *Flaviviridae*. In Brazil, the reemergence and spread of DENV type 4 (DENV-4) across the country were responsible for a significant outbreak in Guarujá, São Paulo, Brazil. Here, we report the first genomic sequences of DENV strains circulating in Guarujá during the 2013 outbreak.

## GENOME ANNOUNCEMENT

Dengue virus is an arbovirus belonging to the genus *Flavivirus* and family *Flaviviridae* (1, 2). The viral particle consists of a positive-sense single-stranded RNA (ssRNA) molecule of approximately 11 kb (3), which encodes three structural and seven nonstructural proteins. The virus is transmitted by mosquitoes of the *Aedes* genus, and it is the causative agent of the dengue fever disease (1). In Brazil, the circulation of the four serotypes after the reemergence of DENV type 4 (DENV-4) in 2010, almost three decades after its last detection, triggered an important emergency situation (4–7). The spread of DENV-4 throughout the country boosted the largest DENV-4 burden in Guarujá, Brazil, in 2013 (8, 9), an important bathing resort, 45 mi from the city of São Paulo, which is the largest metropolis of Brazil. Here, we sequenced the genome from three isolates corresponding to DENV-1 and DENV-4, which were circulating in the 2013 outbreak in Guarujá, São Paulo.

Strains were collected during a previous study in Guarujá (8). Strains GU11 (GU11/2013/BR), GU145 (GU145/2013/BR), and GU128 (GU128/2013/BR) were isolated from serum. Genomic RNA was extracted using the QIAamp viral RNA minikit (Qiagen) according to the manufacturer’s instructions. Following the extraction, the RNA was purified using the RNA Clean* &* Concentrator kit (Zymo Research). Moreover, the RNA quality was checked using NanoDrop (Thermo Fisher), and its concentration was quantified with the Qubit RNA BR assay kit (Invitrogen, Life Technologies, Inc.). The cDNA was obtained using SuperScript III (Invitrogen, Life Technologies, Inc.) and random primers. Likewise, the second strand was synthesized using DNA polymerase I (Invitrogen, Thermo Fisher). The double-stranded cDNA (ds-cDNA) was used as input for library preparation with Nextera XT (Illumina) using the MiSeq platform. The mean sequence quality of the next-generation sequencing (NGS) data was ≥30 (Phred score) for each sample. Adapter and quality trimming were performed with Trim Galore version 0.4.2 (https://www.bioinformatics.babraham.ac.uk/projects/trim_galore/). Filtered paired-end reads were *de novo* assembled with error correction into larger contigs using the SPAdes version 3.5.0 genome assembler (10), under different k-mer values. We obtained three near-fully assembled genomes (i.e., more than 10,000 bp), with an average coverage of more than 150-fold. For each sample, the coding region was reconstructed to its full length.

After a BLAST search (identity, >99%), samples GU11 and GU145 (GenBank accession numbers KY369950 and KY369951, respectively) corresponded to DENV-4 strains, while the sample GU128 was identified as DENV-1 (accession no. KY369949). To corroborate these findings, we reconstructed the phylogenetic relationships via a maximum likelihood (ML) tree with PhyML (11) and using the best-fit model (GTR + G + I). For each sample, we calculated the G+C contents: 47.05% (GU11), 46.92% (GU145), and 46.22% (GU128). The average Shannon entropy showed that DENV-1 (GU128) had the lowest variability (0.01), whereas the DENV-4 genomes (GU11 and GU145) exhibited slightly high levels of variation (0.015 and 0.011, respectively). Furthermore, the selection analysis (*dN*/*dS*) showed evidence of purifying selection acting over the three genomes: 0.090 (GU11), 0.085 (GU145), and 0.050 (GU128).

The reported sequences represent the first available genomes of the largest DENV-4 outbreak in Brazil isolated from São Paulo.

### Accession number(s).

The genome sequences have been deposited at GenBank under accession numbers KY369949 to KY369951.
